# Epigenetic indicators of body mass predict survival outcomes in colorectal cancer patients: patient cohort analysis

**DOI:** 10.1186/s13073-026-01678-y

**Published:** 2026-05-28

**Authors:** Tanwei Yuan, Marko Mandic, Xianzhe Li, Melanie Bewerunge-Hudler, Hermann Brenner, Michael Hoffmeister

**Affiliations:** 1 Division of Clinical Epidemiology of Early Cancer Detection, Molecular Pathological Epidemiology Group, German Cancer Research Center (DKFZ), Im Neuenheimer Feld 581, Heidelberg, 69120 Germany; 2 Medical Faculty Heidelberg, Heidelberg University, Heidelberg, Germany; 3 Microarray Core Facility, German Cancer Research Center (DKFZ), Heidelberg, Germany; 4 Cancer Prevention Graduate School, German Cancer Research Center (DKFZ), Heidelberg, Germany

**Keywords:** Obesity, Epigenetics, Biomarkers, Colorectal cancer, Survival

## Abstract

**Background:**

The prognostic relevance of body mass index (BMI) in colorectal cancer (CRC) remains controversial, partly due to disease-related weight loss. DNA methylation (DNAm)-based BMI scores may be a more stable prognostic indicator than a single BMI measurement. This study evaluated the associations between blood-derived DNAm-BMI scores and mortality in CRC patients, compared with self-reported BMI.

**Methods:**

We analyzed data from 2,126 newly diagnosed CRC patients (41.2% women, median age 69) in the German DACHS study. Self-reported BMI at diagnosis and up to 14 years earlier and pre-diagnostic weight loss was recorded. Five externally developed and validated DNAm-BMI scores were calculated from blood DNAm. Outcomes included all-cause, CRC-specific, and non–CRC-specific mortality. Associations were estimated using Cox proportional hazards models adjusted for demographic, lifestyle, clinical, and treatment factors.

**Results:**

All DNAm-BMI scores showed consistent correlations with self-reported BMI at CRC diagnosis and up to14 years prior (Spearman *r* = 0.15–0.41). Underweight at diagnosis was linked to increased all-cause mortality (adjusted hazard ratio [aHR]: 1.42, 95% confidence interval [CI]: 1.07–1.88), whereas obesity was associated with decreased risk (0.83, 95%CI: 0.70–0.99). In contrast, four of the five DNAm-BMI scores (mBMI-135, mBMI-379, mBMI-435, mBMI-1109) showed consistent, positive linear associations with mortality risk. The 135-CpG score was most predictive (1.57, 95%CI: 1.23–1.99). Most of these associations were confined to the subgroup with blood samples collected before chemo-/radiotherapy.

**Conclusions:**

Blood-based DNAm-BMI scores, reflecting the biological effects of adiposity exposure, were positively associated with CRC mortality, and might be useful to improve risk stratification beyond self-reported BMI.

**Supplementary Information:**

The online version contains supplementary material available at 10.1186/s13073-026-01678-y.

## Background

Excess body fat, commonly measured by body mass index (BMI), is an established risk factor for the development of colorectal cancer (CRC) [1, 2]. However, evidence on the influence of BMI on CRC prognosis remains complex, with mixed findings. Meta-analyses suggest that CRC patients with obesity, or those who are underweight, have poorer long-term survival after surgery, whereas overweight patients may have improved or unchanged survival outcomes [3–5]. Conversely, some studies have reported a survival advantage for overweight or obese individuals at the time of CRC diagnosis [6, 7].

BMI derived from self-reported data at diagnosis may be influenced by disease-related weight changes, [8, 9] introducing bias in assessing the true relationship between obesity and CRC prognosis. One study reported that a BMI decrease of more than five units (in kg/m^2^) in the year before diagnosis was strongly associated with poorer outcomes [7]. To mitigate this issue, another study retrospectively estimated cumulative BMI before diagnosis using self-reported BMI at three time points and a growth curve model [10]. This finding suggested that prolonged exposure to elevated BMI during early to mid-adulthood was associated with worse CRC survival [10]. However, this approach remains vulnerable to recall and measurement bias.

Epigenetic modifications, particularly DNA methylation (DNAm) at CpG sites, capture the cumulative effects of environmental and lifestyle exposures, including obesity, on the genome [11, 12]. Blood DNAm-based biomarkers of BMI have been identified in genes related to lipid metabolism and blood lipid levels, reflecting body fat accumulation and metabolic health [12, 13]. Several DNAm-derived BMI scores have been developed as proxies for chronic exposure to excess weight, [11–15] offering a potential solution to biases introduced by acute weight changes or self-reported inaccuracies. Some of these scores have been associated with poor physical health [12, 13] and an increased risk of several cancers, including CRC [16]. However, their prognostic value in CRC has not yet been investigated.

In this study, we compared associations of CRC prognosis with self-reported BMI at and before diagnosis, pre-diagnostic weight loss and DNAm-based BMI scores. In brief, we used data from the population-based DACHS cohort, including 2,126 CRC patients with baseline blood DNAm data and repeated self-reported weight history. We reconstructed five previously published and externally validated DNAm-based BMI scores and assessed their correlations with self-reported BMI at different time points, as well as their associations with all-cause and cause-specific mortality during a median following of 10.6 years.

## Methods

### Study cohort

We followed the Strengthening the Reporting of Observational Studies in Epidemiology guidelines (STROBE) [17]. This study is based on data from the DACHS (Darmkrebs: Chancen der Verhütung durch Screening, English name: "Colorectal cancer: chances for prevention through screening") cohort, a population-based case‒control and patient cohort study on CRC conducted in 22 hospitals across the Rhine‒Neckar region in southwest Germany between 2003 and 2021. Details of the DACHS study have been described previously [18, 19]. In brief, the participants were German-speaking patients aged ≥ 30 years with a first-time, histologically confirmed primary CRC diagnosis and were physically and mentally capable of completing a one-hour interview. Ethics approval was obtained from the committees of the Medical Faculty of Heidelberg University and the state medical boards of Baden-Wuerttemberg and Rhineland-Palatinate (No. 310/2001).

### Data collection

Baseline data were mostly collected after CRC diagnosis (median: 20 days, IQR: 10–213 days) through structured interviews conducted by trained interviewers. The interviews covered sociodemographic characteristics, lifestyle factors, medical history, and disease symptoms. Information on body weight at each decennial age from 20–80 years and weight and height at diagnosis was obtained via self-reports. Tumor characteristics were extracted from medical records, and disease stage was classified according to the 6th edition of the International Union Against Cancer Tumor, Node, Metastasis (TNM) staging manual [20].

Follow-up began at diagnosis. Standardized data on therapy, comorbidities, and recurrence were collected from physicians at 3, 5, and 10 years postdiagnosis. Vital status, date, and cause of death were retrieved from population registries and public health authorities. A total of 2,126 eligible patients with BMI and blood DNAm data at diagnosis, as well as at least one follow-up assessment, were included in the analysis. The sample size of this study was defined by the largest set of eligible participants within the DACHS cohort.

### DNA methylation preprocessing

Peripheral blood samples were collected post interview and stored at − 80 °C. For the majority of the patients (58%), blood samples were taken within three months of their CRC diagnosis. The majority of samples (78%) were collected after tumor resection surgery. Among the 181 patients who received neoadjuvant therapy, all blood samples were collected after this treatment. Additionally, 500 patients (24%) had blood collected after adjuvant chemotherapy, and 80 patients (3.8%) after adjuvant radiotherapy. DNAm analysis was performed on blood samples from 2126 CRC patients diagnosed between 2003 and 2010 via the Infinium MethylationEPIC BeadChip Kit (Illumina, San Diego, CA, USA, EPIC v1 array), which covers over 850,000 CpG sites. The raw DNAm data files from the iScan array scanner were processed via the 'minfi' R package (version 1.52.1), [21] with missing CpG values imputed via nearest-averaging multiple imputation. Illumina normalization was applied to correct for technical biases and background noise specific to Illumina methylation arrays [21].

### DNAm-based BMI score calculation

We systematically identified previously developed blood-based DNAm-based scores predictive of BMI through a literature search of PubMed using keywords including '(epigenetic OR DNA methylation) AND score AND (BMI OR "body mass index")'. Scores were eligible for inclusion if they were: 1) developed and validated in at least one external cohort; and 2) the full list of CpG sites and their corresponding regression coefficients was publicly available, allowing reconstruction via linear combination (Score = Intercept + Σ[CpG × coefficient]).

Five scores met our eligibility criteria [11–15]. All were developed by identifying CpG sites whose methylation levels were associated with self-reported or measured BMI, typically using epigenome-wide association analyses. These CpGs were then combined into weighted prediction models using different statistical approaches. Two scores were developed using least absolute shrinkage and selection operator (LASSO) regression, [11, 13] two used Elastic Net regression, [14, 15] and one used meta-analyzing CpG-specific coefficients estimated from linear regression models across three cohorts [12].

**Fig. 1:**
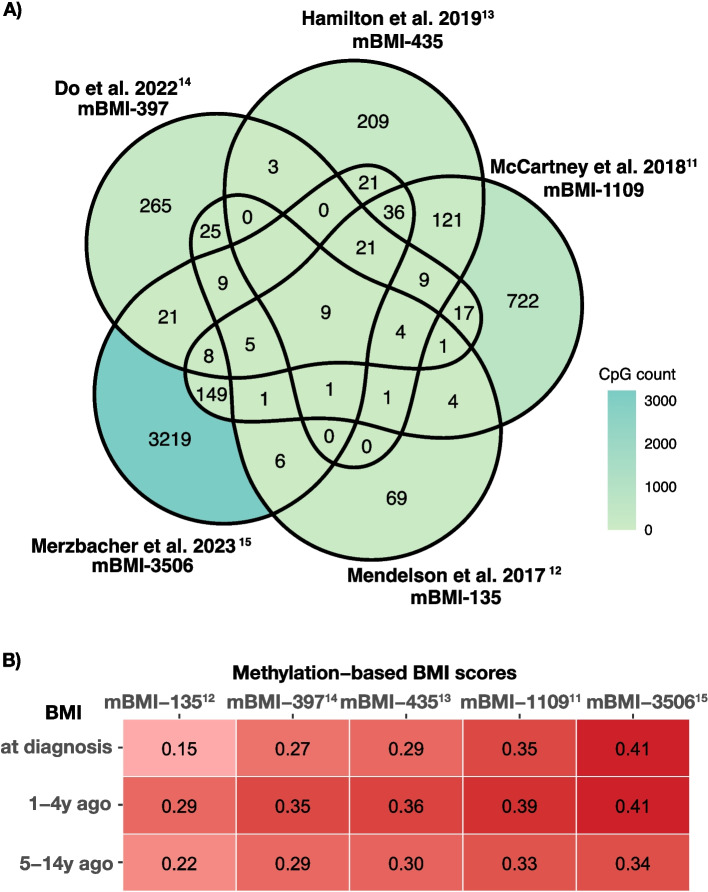
**a** Overlapping CpGs of proposed blood methylation-based BMI scores. **b** Spearman correlation coefficients between blood DNAm-based BMI scores and self-reported BMI at different time periods. mBMI = methylation-based BMI, DNAm = DNA methylation. The number after mBMI stands for the number of CpGs in the score, and the superscript represents the reference of the paper that developed this score. For the Spearman correlation coefficients, all P values were < 0.0001

The number of CpGs included in each score ranged from 135 (Mendelson et al. [12]) to 3506 (Merzbacher et al. [15]). Most CpGs were unique to each score, with only nine CpGs common to all scores (Fig. 1A). The full list of CpGs and coefficients used to calculate each score in our cohort is provided in Additional file 1: Supplementary Data.

### Statistical analysis

Sociodemographic and clinical variables were summarized via descriptive statistics and compared between included and excluded patients. The median follow-up time was estimated via the reverse Kaplan‒Meier method [22]. Missing values for patient characteristics adjusted as covariates in the multivariable Cox regression model were handled via multiple imputations via chained equations, using the 'mice' R package (version 3.17.0), [23] with weight at each decennial age and death outcome (i.e., the Nelson–Aalen estimator of the baseline cumulative hazard and the outcome indicator) [24] being included in the imputation procedure. All analyses were conducted in parallel across 20 imputed datasets, with the results combined via Rubin's rule [25].

Self-reported weight was recorded at each decennial age (20, 30, 40, 50, 60, 70, and 80 years) prior to CRC diagnosis. Associations between five methylation-based BMI scores and self-reported BMI at diagnosis, 1–4 years before diagnosis, and at least five years before diagnosis (ranging from 5–14 years prior, Additional file 2: Table S1) were assessed via Spearman correlation coefficients. To evaluate whether these correlations were independent of the temporal stability of BMI, we additionally restricted the correlation analysis to the subgroup of patients who experienced substantial pre-diagnostic weight change, defined as a > 5 kg change in body weight between diagnosis and 5–14 years prior.

To assess the impact of pre-diagnostic weight loss on prognosis, we calculated the weight change between diagnosis and at least five years prior. Cox proportional hazards models were used to evaluate associations between self-reported BMI at diagnosis, BMI 1–4 years before diagnosis, BMI 5–14 years before diagnosis, pre-diagnostic weight loss, five blood methylation-based BMI scores, and all-cause mortality. To distinguish the independent effects of BMI from weight change, additional Cox models simultaneously included BMI (at diagnosis or 5–14 years prior) and pre-diagnostic weight loss in the same model.

The proportional hazards assumption was assessed via scaled Schoenfeld residuals. To account for the interval between CRC diagnosis and blood sample collection, delayed-entry Cox models were used [26]. Cause-specific Cox proportional hazards models were applied for CRC-specific and non-CRC-related mortality, with death from other causes treated as competing events.

Cox models were adjusted for age, sex, tumor stage, tumor location, treatment with neoadjuvant therapy, treatment with chemotherapy or radiotherapy, alcohol consumption, physical activity, smoking status, regular statin use, nonsteroidal anti-inflammatory drug use, history of cardiovascular diseases, hyperlipidemia, hypertension, and other cancers. BMI was modeled both continuously (per 5-unit increase) and categorically (BMI < 20, 20–25, 25–30, > 30). Weight change was categorized as > 5 kg loss, 2–5 kg loss, or no weight loss. Methylation-based BMI scores were standardized, with one-unit increase corresponding to one standard deviation, and categorized into quartiles, with the lowest quartile as a reference. Linear trends across quartiles were tested by modeling the quartile median as a continuous variable in the Cox model.

To assess dose‒response relationships, we used restricted cubic spline functions with three knots to examine the associations between self-reported BMI, weight change, or methylation-based BMI scores and mortality. We reported both the *P* value for overall linearity and the *P* value for curvature to assess the shape of each dose–response.

Subgroup analyses were conducted by BMI categories, weight loss categories, median age (≤ 69 vs. > 69 years), sex, TNM stage (I/II vs. III/IV), tumor metastasis, tumor location (distal colon, proximal colon, or rectum). To assess the influence of blood collection timing, we conducted further analyses based on the interval from CRC diagnosis to blood draw (< 3 months vs. ≥ 3 months), and the timing relative to systemic therapy: before or without versus after neo-/adjuvant chemotherapy, and before or without versus after chemo-/radiotherapy. For each subgroup analysis, a *P* value for interaction was calculated to statistically test for effect modification.

### Gene ontology enrichment analysis

Given the limited functional annotation of the five DNAm-based BMI scores in the original publications [11–15], we performed Gene Ontology (GO) enrichment analysis to interpret their biological relevance. For each of the five epigenetic scores, the list of CpG sites included in the score was extracted and mapped to their corresponding genes using the UCSC Reference Gene annotation (based on the GRCh37/hg19 genome build) from the 'IlluminaHumanMethylationEPICanno.ilm10b4.hg19' package (version 0.6.0) [27, 28].

For each gene list, we identified unique protein-coding genes, collapsing multiple transcripts to a single gene identifier. These gene symbols were then converted to Entrez Gene IDs using the 'org.Hs.eg.db' R package (version 3.20.0) [29]. GO enrichment analysis for biological processes was carried out using the 'enrichGO' function from the 'clusterProfiler' R package (version 4.14.6) [30]. The analysis was performed with a significance threshold of an adjusted *P* value < 0.05 (Benjamini–Hochberg method) and a *q* value cutoff of 0.2 [31].

Statistical significance was set at a two-sided *P* value < 0.05. All analyses were performed via R version 4.4.0.

## Results

### Characteristics of the study cohort

The flow chart detailing study population selection is shown in Additional file 2: Fig. S1. Additional file 2: Table S2 compares baseline characteristics between the 2126 included and 4476 excluded patients, showing that the two groups were comparable across key factors including age, sex, BMI, smoking status, lifetime alcohol consumption, history of diabetes, hypertension, hyperlipidemia, and use of nonsteroidal anti-inflammatory drugs, supporting the representativeness of the analytic sample.

Baseline characteristics of the study population are summarized in Table 1. The median age at diagnosis was 69 years (interquartile range [IQR]: 62–77), and 40.9% of the patients were female. The majority of patients (68.8%) had fewer than nine years of schooling. Most patients had stage II (34.8%) or III (33.2%) CRC. Nearly half of the patients (48.4%) received chemotherapy or radiotherapy following surgery. The median BMI at diagnosis was 26.2 kg/m^2^, with 42.1% classified as overweight (BMI 25–30) and 19.0% classified as obese (BMI ≥ 30). The median follow-up duration was 10.6 years (IQR: 10.1–14.4). During follow-up, 1171 patients (55.1%) died from all causes, including 600 deaths attributed to CRC.

**Table 1:** Baseline characteristics of the study population

Characteristics	Blood sample No. (%) (*N* = 2126)	Missing
Median age (IQR)	69.0 (62.0, 77.0)	–
Female	869 (40.9)	–
Schooling years		
< 9	1463 (68.8)	4 (0.2)
9–10	350 (16.5)	
> 10	309 (14.5)	
Body mass index kg/m^2^		
Median (IQR)	26.2 (23.7, 29.0)	–
< 20	100 (4.7)	
20–25	727 (34.2)	
25–30	894 (42.1)	
≥ 30	405 (19.0)	
TNM stage		11 (0.5)
I	379 (17.8)	
II	739 (34.8)	
III	705 (33.2)	
IV	292 (13.7)	
Neoadjuvant therapy	181 (8.5)	1 (0.05)
Adjuvant chemotherapy or radiotherapy	1030 (48.4)	3 (0.1)
Tumor location		–
Distal colon	590 (27.8)	
Proximal colon	771 (36.3)	
Rectum	764 (35.9)	
Smoking status		3 (0.1)
Never	976 (45.9)	
Former	819 (38.5)	
Current	328 (15.4)	
Lifetime alcohol consumption^a^		6 (0.3)
Abstainers	362 (17.0)	
Light drinkers	1194 (56.2)	
Moderate drinkers	403 (19.0)	
Heavy drinkers	161 (7.6)	
Physical activity^b^, median (IQR)	191.1 (130.6, 278.1)	34 (1.6)
Use of statins	312 (14.7)	2 (0.1)
Use of NSAIDs	529 (24.9)	–
History of diabetes	396 (18.6)	12 (0.6)
History of hypertension	1097 (51.6)	23 (1.1)
History of hyperlipidemia	655 (30.8)	91 (4.3)
History of cardiovascular diseases	569 (26.8)	61 (2.9)
History of other cancers	232 (10.9)	10 (0.5)

*IQR* Interquartile range, *TNM* Tumor, lymph node, and metastasis staging system, *NSAIDs* Nonsteroidal anti-inflammatory drugs

^a^Sex-specific categories of abstainers, light, moderate, or heavy drinkers: for women, 0, > 0–12, > 12–25, or > 25 g ethanol/day; for men, 0, > 0–24, > 24–50, or > 50 g ethanol/day

^b^Lifetime average metabolic equivalent of task hours/week

### Associations between DNAm-derived and self-reported BMI scores

All five blood DNAm-based BMI scores were consistently correlated with self-reported BMI at diagnosis and up to 14 years before diagnosis (all *P* < 0.0001; Fig. 1B). As expected, given that these scores were originally derived using contemporaneous or recent BMI measurements, the strongest correlation was observed for BMI measured 1–4 years before diagnosis (median Spearman correlation: 0.36, range: 0.29–0.41). The correlations only slightly decreased for BMIs measured 5–14 years before diagnosis (0.30, 0.22–0.34), likely reflecting natural weight changes over time rather than loss of epigenetic signal. In the subgroup of patients (*N* = 936) with substantial pre-diagnostic weight change (> 5 kg), the correlations with BMI 1–4 years prior were slightly stronger (median 0.38, 0.30–0.46), and correlations with BMI 5–14 years prior remained highly significant with only minimal attenuation (0.28, 0.19–0.29; Additional file 2: Fig. S2).

Scores incorporating a greater number of CpG sites tended to have higher correlation coefficients, which was expected given the larger number of variables contributing to the score. For example, the score developed by Merzbacher et al. [15]. The presence of 3506 CpGs was most strongly associated with self-reported BMI at and before diagnosis (*r* = 0.34–0.41), whereas the score from Mendelson et al. [12]. A total of 135 CpGs had slightly lower correlations, peaking at 0.29.

### Associations of self-reported and methylation-based BMI scores with mortality risk

**Fig. 2:**
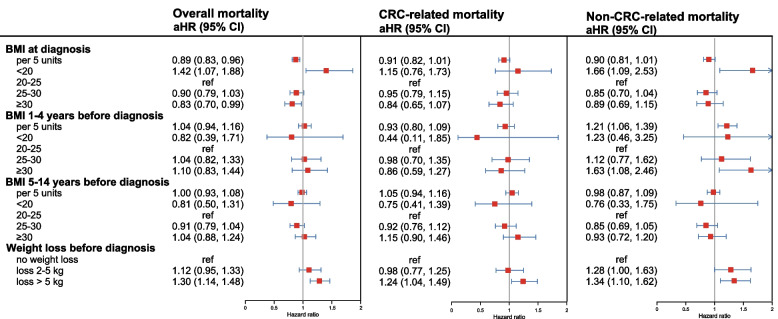
Associations between self-reported BMI and weight loss with CRC prognosis Weight loss was calculated as the difference between weight at diagnosis and weight 5–14 years before diagnosis. The multivariable Cox model was adjusted for age, sex, tumor stage, tumor location, neoadjuvant therapy, adjuvant treatment with chemotherapy or radiotherapy, alcohol consumption, physical activity, smoking status, regular statin use, nonsteroidal anti-inflammatory drug use, history of cardiovascular diseases, hyperlipidemia, hypertension, and other cancers. CRC = colorectal cancer, aHR = adjusted hazard ratio, CI = confidence interval

In the overall study cohort (Fig. 2), being underweight (BMI < 20) at diagnosis was associated with an increased risk of overall mortality (adjusted hazard ratio [aHR]: 1.42, 95% confidence interval [CI]: 1.07–1.88) and non-CRC-related mortality (1.66, 1.09–2.53). Conversely, obesity (BMI ≥ 30) at diagnosis was associated with a slightly lower risk of overall mortality (0.83, 0.70–0.99). A five-unit increase in BMI at diagnosis was associated with an 11% reduced risk of overall mortality (0.89, 0.83–0.96). In contrast, being obese 1–4 years before CRC diagnosis was associated with an increased risk of non-CRC-related mortality (1.63, 1.08–2.46), and each five-unit increase in BMI during this period was associated a 21% higher risk of non-CRC-related mortality (1.21, 1.06–1.39). No significant associations were observed for BMI measured 5–14 years before diagnosis. Notably, weight loss of more than 5 kg at CRC diagnosis compared with 5–14 years before CRC diagnosis was strongly associated with increased overall (1.30, 1.14–1.48), CRC-related (1.24, 1.04–1.49), and non-CRC-related (1.34, 1.10–1.62) mortality.

When BMI (at diagnosis or 5–14 years prior) and weight loss were simultaneously included in the same Cox models (Additional file 2: Table S3), the increased mortality risk associated with being underweight at diagnosis was modestly attenuated but remained statistically significant. In contrast, the modest protective association observed for obesity at diagnosis was substantially attenuated and no longer statistically significant. Associations between BMI measured 5–14 years before diagnosis and mortality remained non-significant after accounting for weight loss.

**Fig. 3:**
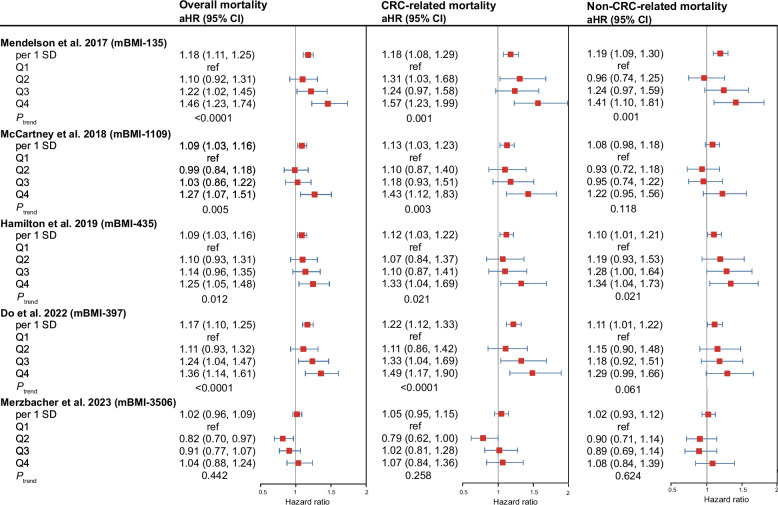
Associations between methylation-based BMI scores and CRC prognosis The multivariable Cox model was adjusted for age, sex, tumor stage, tumor location, neoadjuvant treatment, adjuvant treatment with chemotherapy or radiotherapy, alcohol consumption, physical activity, smoking status, regular statin use, nonsteroidal anti-inflammatory drug use, history of cardiovascular diseases, hyperlipidemia, hypertension, and other cancers. CRC = colorectal cancer, aHR = adjusted hazard ratio, CI = confidence interval. mBMI = methylation-based BMI. The number after mBMI stands for the number of CpGs included in the score

In contrast, four of the five DNAm-based BMI scores were positively associated with mortality risk, with higher scores corresponding to greater mortality hazard values (Fig. 3). The strongest association was observed for the 135-CpG score from Mendelson et al. [12], which was consistently associated with an increased risk of overall mortality (highest vs. lowest quartile: 1.46, 1.23–1.74, *P*_trend_ < 0.0001), CRC-related mortality (1.57, 1.23–1.99, *P*_trend_ = 0.001), and non-CRC-related mortality (1.41, 1.10–1.81, *P*_trend_ = 0.001). A similarly strong association was found for the 397-CpG score from Do et al. [14] and overall mortality (1.36, 1.14–1.61, *P*_trend_ < 0.0001), CRC-related mortality (1.49, 1.17–1.90, *P*_trend_ < 0.0001), and non-CRC-related mortality (1.29, 0.99–1.66, *P*_trend_ = 0.061).

The 435-CpG score from Hamilton et al. [13] also showed significant associations with the three types of mortality risk, and the 1109-CpG score from McCartney et al. [11] was significantly associated with overall and CRC-specific mortality. Additionally, when the categorical quartiles of these four scores were examined, [11–14] higher quartiles were consistently associated with stepwise increased mortality risk. However, the 3506-CpG [15] score from Merzbacher et al. was not significantly associated with mortality outcomes. The only exception was a modest inverse association between the second quartile of this score and overall mortality (0.82, 0.70–0.97).

### Dose‒response analyses

**Fig. 4:**
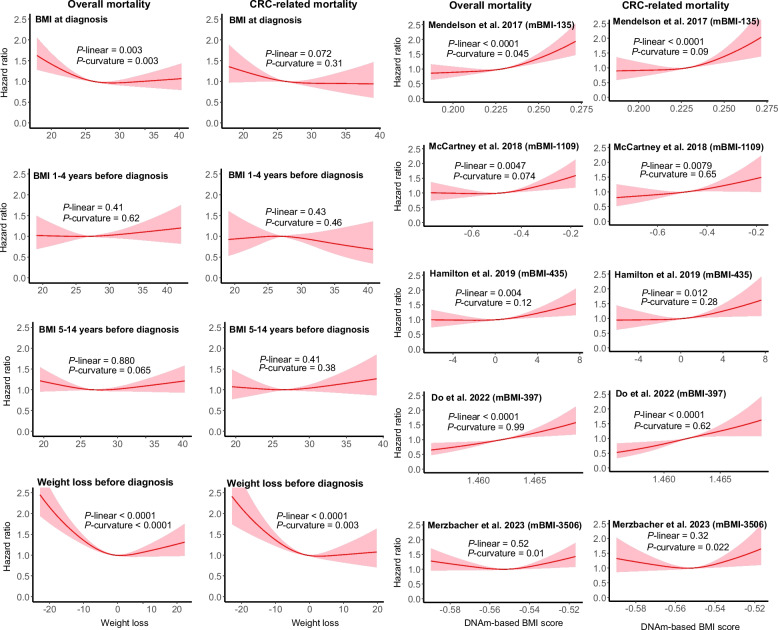
Dose‒response relationships between self-reported BMI and methylation-based BMI scores and overall mortality and CRC-related mortality. Weight loss was calculated as the difference between weight at diagnosis and weight 5–14 years before diagnosis. The dose–response curves were adjusted for age, sex, tumor stage, tumor location, neoadjuvant therapy, adjuvant treatment with chemotherapy or radiotherapy, alcohol consumption, physical activity, smoking status, regular statin use, nonsteroidal anti-inflammatory drug use, history of cardiovascular diseases, hyperlipidemia, hypertension, and other cancers. BMI = body mass index; mBMI = methylation-based BMI. The number after mBMI stands for the number of CpGs included in the score

The dose‒response analysis (Fig. 4) provided further insight into the shape of the associations. For self-reported BMI at diagnosis, we observed a modest U-shaped relationship with overall mortality risk, supported by significant *P* values for both linearity and curvature. In contrast, BMI measured 1–4 years or 5–14 years prior to diagnosis showed no clear association with overall or CRC-related mortality, with both the linear and curvature tests suggesting an essentially flat relationship.

For weight change 5–14 years before diagnosis, the dose–response curve demonstrated a strong non-linear association with mortality. Substantial weight loss was associated with a steep increase in risk, and substantial weight gain showed a more moderate elevation, with significant *P* values for both linearity and curvature confirming this pronounced U-shaped pattern.

In contrast, four methylation-based BMI scores [11–14] exhibited predominantly positive linear association of higher scores with overall mortality and CRC-related mortality, reflected by significant *P* values for linearity and little evidence of curvature. However, for the 3506-CpG score from Merzbacher et al., [15] a U-shaped shape pattern was observed, supported by significant curvature testing, showing a modestly elevated risk at both lower and higher score levels.

### Subgroup analyses

In subgroup analyses stratified by BMI at diagnosis and weight loss, the statistically significant positive associations between methylation-based score [11–14] with mortality were similar across subgroups (Additional file 2: Table S4). Further subgroup analyses by patient characteristics (Additional file 2: Table S5) demonstrated consistent positive associations between the four methylation-based scores [11–14], particularly the two scores from Mendelson et al. [12] and Do et al. [14], and mortality outcomes across various patient subgroups. These associations remained robust among patients aged < 70 years or ≥ 70 years, both sexes, those with stage I/II or III/IV CRC, individuals with metastatic or nonmetastatic disease, those with tumors located proximal to the colon, distal colon, or rectum. Additionally, the associations persisted regardless of whether blood samples were collected within three months or later after CRC diagnosis.

Most subgroup variables did not show statistically significant interaction with either the self-reported or the DNAm-based BMI scores.

The positive associations between higher DNAm-based BMI scores and increased mortality risk were largely confined to patients whose blood was collected before any chemotherapy or chemo-/radiotherapy, or who never received systemic treatment, except for the mBMI-397 score [14] which remained positively associated with CRC-specific mortality even among patients whose blood was drawn after chemotherapy (1.25, 1.09–1.43) or after chemo-/radiotherapy (1.24, 1.08–1.42).

### Gene ontology enrichment analysis

We performed GO enrichment analyses for the CpG sites included in each DNAm-BMI score to explore the underlying biological pathways. The 135-CpG score from Mendelson et al. [12] was enriched for pathways involved in response to nutrients and extracellular stimuli, interferon-gamma production, and JAK-STAT signaling (Additional file 2: Fig. S3). Similarly, the 397-CpG score [14] showed enrichment for cell migration, T-helper 2 immune differentiation, and glycolytic processes (Additional file 2: Fig. S4).

The 435-CpG score [13] was enriched in cell adhesion pathways, vascular development including angiogenesis, and phospholipid homeostasis (Additional file 2: Fig. S5). The 1109-CpG score [11] showed enrichment in cell adhesion, cognition and synapse organization, nutrient and oxygen response including hypoxia, epithelial tube morphogenesis, embryonic organ development, and axonogenesis (Additional file 2: Fig. S6). Finally, the Merzbacher et al. score, [15] which comprises the largest number of CpGs, exhibited the broadest and most diverse functional landscape. This included cell adhesion, neurodevelopment, growth factor signaling (TGF-β, FGF), Wnt pathway activation, and extensive morphogenetic processes, reflecting complex and multifaceted biological regulation (Additional file 2: Fig. S7).

## Discussion

In this large cohort study, all five blood-derived DNAm-based BMI scores were consistently correlated with both self-reported current BMI and in the years prior to diagnosis. Their modest magnitudes suggest that these scores capture BMI-related, but not BMI-identical, biological information. Patients who were underweight at diagnosis (BMI < 20) or had experienced weight loss of more than 5 kg at least five years before diagnosis had poorer prognostic outcomes, whereas obesity was modestly associated with reduced mortality risk. In contrast, four [11–14] of the five methylation-based BMI scores were significantly associated with increased overall and CRC-related mortality, and three [12–14] were additionally associated with non-CRC-related mortality. Interestingly, we found that the mBMI-135 score, which showed the weakest correlation with BMI at diagnosis, demonstrated the strongest and most consistent associations with all mortality outcomes. These associations remained robust across major clinical subgroups and were most evident in patients whose blood samples were collected before chemo-/radiotherapy.

Our findings confirmed that these five previously developed blood methylation-based BMI scores are significantly correlated with self-reported BMI. Some of these scores showed similar results in other cohorts [13, 16, 32]. However, we provide the first evidence that these scores not only correlate with BMI at the time of blood sample collection but also consistently reflect BMI up to 14 years prior to diagnosis. Importantly, these correlations persisted and remained highly significant even among patients who experienced substantial pre-diagnostic weight change, supporting their stability in reflecting the biological effect of adiposity exposure under the condition of rapid weight fluctuation. These findings suggest that epigenetic modifications induced by obesity may accumulate over time. Indeed, a recent study demonstrated that adipose tissue retains cellular transcriptional changes after weight loss in both humans and mice, suggesting a form of epigenetic memory related to obesity [33]. We also found that scores incorporating a greater number of CpG sites showed slightly stronger associations with BMI at various time points, which is expected, given that larger numbers of CpGs likely provide a more comprehensive reflection of BMI.

Previous studies have reported an “obesity paradox,” where a higher BMI at cancer diagnosis is associated with improved survival, whereas underweight patients experience significantly worse outcomes [6, 7, 34]. The inverse relationship between self-reported BMI and mortality risk observed in our study aligns with these findings. One possible explanation is that higher body weight may serve as a nutritional reserve, potentially benefiting patients experiencing the physiological stress of cancer treatment or disease progression [35]. However, the protective effect of obesity at diagnosis in our study was minimal and became attenuated after accounting for pre-diagnostic weight loss. This suggests that the modest protective may be confounded by the fact that patients with higher pre-diagnostic BMI had greater capacity for weight loss, which itself is a strong indicator of poor prognosis. The finding that obesity 1–4 years before diagnosis was associated with a higher risk of non-CRC-related mortality likely reflects the unmasked detrimental effect of excess weight on metabolic and cardiovascular health, increasing vulnerability to death from non-cancer causes. The stark reversal of this risk association by the time of diagnosis might reflect how acute, disease-driven weight loss can obscure the true relationship between chronic obesity and survival.

In contrast, the increased mortality risk observed among underweight patients at diagnosis remained statistically significant even after adjustment for weight loss. This suggests that the detrimental effect of being underweight at diagnosis is not solely driven by weight loss, supporting the possibility of reverse causality where low BMI may result from underlying illness or disease-related cachexia rather than being a direct cause of increased mortality [34, 36]. Indeed, no significant associations were observed between BMI measured 5–14 years prior to diagnosis and mortality risk, contrasted with the strong adverse effects of > 5 kg pre-diagnostic weight loss. This finding is consistent with a previous study based on an earlier dataset of the DACHS cohort, with fewer participants [7].

These findings suggest that the relationship between BMI and CRC prognosis is likely confounded by disease-related weight loss, making BMI at diagnosis an incomplete indicator of cumulative excess weight. Moreover, BMI at diagnosis cannot reflect body composition complexity, as not all adipose tissue is metabolically equivalent [37, 38]. For example, visceral adiposity is strongly linked to systemic inflammation, insulin resistance, and more aggressive CRC biology, [38, 39] whereas subcutaneous fat may be more metabolically benign and potentially serve as a nutritional reserve during illness [37, 38]. Previous research has highlighted that self-reported BMI may overestimate mortality risk in underweight patients, [36] further questioning its reliability as a prognostic measure. This underscores the limitations of using a single BMI measurement at diagnosis to capture the complex and nuanced interplay between body composition, weight trajectories, and health outcomes in CRC patients [36].

Our study revealed significant associations between four of the five examined DNAm-based BMI scores and mortality risk in CRC patients, with the strongest associations observed for the mBMI-135 [12] and mBMI-397 scores [14]. The consistent significant associations of these scores across different patient subgroups further corroborated their prognostic value. Interestingly, the two scores, especially the mBMI-135, showed relatively weak associations with self-reported BMI. This suggests their prognostic power may depend less on approximating current BMI and more on capturing broader biological states related to metabolic dysregulation. Indeed, results from our GO analysis revealed that the two scores are enriched in pathways related to nutrient and inflammatory signaling, immune regulation, lipid metabolism, and cellular stress responses. These pathways are central to obesity-related metabolic perturbation, and have been implicated in CRC progression, treatment response, and survival [40, 41]. For example, the CpG site cg06690548 in *SLC7A11* gene, [42] a locus shared across all scores, which plays a role in oxidative stress resistance and has been associated with advanced tumor stage and poorer CRC prognosis [43]. Our results suggest that these scores may serve as more reliable indicators of metabolic health related to excess body fat, capturing the biological consequences of obesity that contribute to CRC prognosis.

In contrast, the 3506-CpG score from Merzbacher et al. [15] did not show a clear pattern of associations with mortality outcomes in the overall cohort. But its prognostic relevance emerged among patients with metastasis. This score includes the largest number of CpGs and showed the strongest correlation with self-reported BMI among the five scores, indicating that it captures a broad spectrum of adiposity-related methylation patterns. However, its extensive CpG coverage may dilute the influence of CpG sites directly involved in cancer-relevant biological pathways, reducing its prognostic sensitivity in the general CRC population. In patients with metastasis, metabolic dysregulation, inflammation, and treatment-induced biological stress are more pronounced, potentially amplifying the methylation signals captured by this comprehensive score. These findings underscore that the clinical value of DNAm-BMI scores may not lie in their ability to approximate BMI, but in their capacity to capture biologically meaningful obesity-related processes relevant to CRC prognosis.

Our subgroup analyses showed that most DNAm-BMI scores were positively associated with mortality mainly when blood samples were collected before chemo-/radiotherapy or in patients who never received systemic treatment. This is biologically plausible, as chemotherapy and radiotherapy induce epigenetic changes reflecting treatment-related stress or accelerated aging rather than adiposity [44, 45], potentially confounding post-treatment methylation profiles. In contrast, the mBMI-397 score [14] remained associated with CRC-specific mortality even after treatment. Our GO analysis suggests this exception may be explained by the score's aggregation of signals from pathways central to aggressive disease, such as cell migration, pro-tumorigenic immune responses (e.g., T-helper 2 differentiation), and glycolytic metabolism, which might be more resilient to therapy-induced confounding. These findings highlight the necessity of considering treatment timing in epigenetic prognostic studies and support measuring DNAm-BMI scores prior to systemic therapy for more accurate risk stratification in CRC patients.

This study has several limitations. First, BMI data were self-reported and therefore susceptible to recall bias. Second, we lacked data on the waist‒to-hip ratio, a more precise indicator of intra-abdominal fat accumulation that may better predict health risks than BMI [46, 47]. However, we assessed the associations between median or mean BMI from age 20–14 years before CRC diagnosis and lifetime excess weight and mortality risk, but none of these associations were statistically significant (data not shown). Third, blood samples for DNAm measurement were not collected at the same time for all patients. Nevertheless, we were able to perform subgroup analyses stratified by blood collection timing relative to CRC diagnosis and chem-/radiotherapy. Fourth, while we adjusted for a wide range of potential confounders, residual confounding from unmeasured metabolic factors cannot be ruled out. Finally, our study population was restricted to CRC patients from Germany, and further research is needed to assess the generalizability of our findings to other populations.

The clinical implications of our findings are noteworthy. DNAm-based BMI scores, particularly those with strong and consistent associations with mortality, may serve as valuable biomarkers for risk stratification in CRC patients. Unlike self-reported BMI, which reflects body weight at a single time point and is subject to measurement errors and drastic weight changes, methylation-based scores stably capture biological effects linked to excess body fat and metabolic dysfunction. This dynamic measure of metabolic health could enhance clinical decision-making by identifying patients at elevated risk of mortality owing to sustained obesity-related biological disruptions, even in the absence of overt obesity at diagnosis. This integrative measure of metabolic health could help identify patients at elevated mortality risk owing to sustained obesity-related biological disruptions, even in the absence of overt obesity at diagnosis, and may inform closer surveillance, personalized treatment strategies (e.g., therapies targeting insulin resistance), or targeted lifestyle interventions. Nevertheless, estimating DNAm-based scores requires additional blood-based assays and computational resources, which may currently limit routine clinical implementation. Future studies could directly compare the prognostic value of these epigenetic scores against imaging-derived metrics like visceral adiposity [48] to determine if they offer independent or synergistic information. Validating these scores in diverse populations and evaluating their integration into multi-modal clinical workflows will be essential next steps.

## Conclusions

In conclusion, our study demonstrated that blood-derived DNAm-based BMI scores offer a robust, biologically informed alternative to traditional BMI for assessing obesity-related mortality risks in CRC patients. These scores circumvent the limitations of self-reported measures by encapsulating the enduring metabolic and epigenetic consequences of adiposity, thereby providing a more nuanced tool for prognosis and personalized care. While further validation is needed, our findings underscore the promise of epigenetics in bridging the gap between epidemiological observations and clinical practice in oncology.

## Supplementary Information

Additional file 1. List of all CpG sites and corresponding coefficients required to calculate the five DNAm-based BMI scores used in this study.

Additional file 2. Supplementary figures and tables.

## Data Availability

All analysis code is publicly available on GitHub: https://github.com/TanweiY/BMIepigenetic. Due to Institutional Review Board restrictions and participant confidentiality requirements, raw DNA methylation and patient-level data cannot be made public. However, the data are archived within the DACHS study database at the German Cancer Research Center (DKFZ) and can be made available to researchers under a formal collaboration agreement. Data access requests, including a detailed study protocol and request for collaboration, should be submitted to the corresponding author (m.hoffmeister@dkfz.de) and will be reviewed by the DACHS Data Access Committee. Approved requests will be processed within approximately one month, and data will be provided in a secure manner in compliance with ethical and data protection regulations.

## Abbreviations

BMI: Body mass index
CRC: Colorectal cancer
DNAm: DNA methylation
aHR: Adjusted hazard ratio
DACHS: Darmkrebs: Chancen der Verhütung durch Screening, English name: “Colorectal cancer: chances for prevention through screening”
TNM: Tumor, Node, Metastasis staging system
