# Supplementary material for: Epigenetic indicators of body mass predict survival outcomes in colorectal cancer patients: patient cohort analysis
Source: Genome Med. 2026 May 28;18:73. doi: 10.1186/s13073-026-01678-y (PMC13218036; doi:10.1186/s13073-026-01678-y)
Supplement: Supplementary file 2 — Additional file 2. Supplementary figures and tables. [file 13073_2026_1678_MOESM2_ESM.docx]

Study population

(n = 2126)

Patients without baseline BMI

(n = 10)

Patients without follow-up assessment

(n = 1)

Patients without blood DNA methylation measurement after CRC diagnosis

(n = 4465)

**Fig. S1: Flow chart of study population**

Total CRC patients included in the DACHS study at baseline

(n = 6602)

**Table S1: Patient groups with weight measured at least five years ago (ranging from 5-14 years)**

| Age at weight measurement | Patient groups |
| --- | --- |
|  | Age at diagnosis |
| 20 | 25, 26, 27, 28, 29, 30, 31, 32, 33, 34, |
| 30 | 35, 36, 37, 38, 39, 40, 41, 42, 43, 44 |
| 40 | 45, 46, 47, 48, 49, 50, 51, 52, 53, 54 |
| 50 | 55, 56, 57, 58, 59, 60, 61, 62, 63, 64 |
| 60 | 65, 66, 67, 68, 69, 70, 71, 72, 73, 74 |
| 70 | 75, 76, 77, 78, 79, 80, 81, 82, 83, 84 |
| 80 | 85, 86, 87, 88, 89, 90, 91, 92, 93, 94 |

Weights was measured at each decennial age (20, 30, 40, 50, 60, 70, and 80 years).

**Table S2: Comparison of patient characteristics between included and excluded patients**

| **Characteristics** | **Included patients (N = 2126)** | **Excluded patients (N = 4476)** | ***P* value** |
| --- | --- | --- | --- |
| Median age (IQR) | 69.0 (62.0, 77.0) | 70.0 (61.0, 77.0) | 0.946 |
| Female | 869 (40.9) | 1809 (40.4) | 0.743 |
| Schooling years |  |  |  |
| <9 | 1463 (68.9) | 2751 (61.6) | <0.001 |
| 9-10 | 350 (16.5) | 827 (18.5) |  |
| >10 | 309 (14.6) | 890 (19.9) |  |
| Missing | 4 | 8 |  |
| Body mass index kg/m2 |  |  |  |
| Median (IQR) | 26.2 (23.7, 29.0) | 26.0 (23.6, 29.3) | 0.706 |
| <20 | 100 (4.7) | 228 (5.1) | 0.41 |
| 20-25 | 727 (34.2) | 1524 (34.3) |  |
| 25-30 | 894 (42.1) | 1787 (40.2) |  |
| ≥30 | 405 (19.0) | 903 (20.3) |  |
| Missing | 0 | 34 |  |
| TNM stage |  |  |  |
| I | 379 (17.9) | 1129 (25.5) | <0.001 |
| II | 739 (34.9) | 1248 (28.2) |  |
| III | 705 (33.3) | 1374 (31.1) |  |
| IV | 292 (13.8) | 671 (15.2) |  |
| Missing | 11 | 54 |  |
| Neoadjuvant therapy | 181 (8.5) | 542 (12.1) | <0.001 |
| Missing | 1 | 1124 |  |
| Adjuvant chemotherapy or radiotherapy | 1513 (45.2) | 1030 (48.5) | 0.017 |
| Missing | 3 | 1127 |  |
| Tumor Location |  |  |  |
| Distal colon | 590 (27.8) | 1190 (26.6) | 0.006 |
| Proximal colon | 771 (36.3) | 1495 (33.4) |  |
| Rectum | 764 (36.0) | 1789 (40.0) |  |
| Missing | 1 | 2 |  |
| Smoking status |  |  |  |
| Never | 976 (46.0) | 1970 (44.4) | 0.492 |
| Former | 819 (38.6) | 1767 (39.9) |  |
| Current | 328 (15.4) | 697 (15.7) |  |
| Missing | 3 | 42 |  |
| Lifetime alcohol consumption^1^ | | | |
| Abstainers | 362 (17.1) | 859 (19.3) | 0.06 |
| Light drinkers | 1194 (56.3) | 2511 (56.5) |  |
| Moderate drinkers | 403 (19.0) | 784 (17.6) |  |
| Heavy drinkers | 161 (7.6) | 294 (6.6) |  |
| Missing | 6 | 28 |  |
| Physical activity^2^, median (IQR) | 191.1 (130.6, 278.1) | 176.1 (123.1, 237.6) | <0.001 |
|  | 34 | 51 |  |
| History of cardiovascular diseases | 569 (27.6) | 979 (22.7) | <0.001 |
| Missing | 61 | 164 |  |
| Use of statins | 312 (14.7) | 901 (20.1) | <0.001 |
| Missing | 2 | 1 |  |
| Use of NSAIDS | 529 (24.9) | 1104 (24.7) | 0.872 |
| History of diabetes | 396 (18.7) | 863 (19.4) | 0.52 |
| Missing | 12 | 36 |  |
| History of hypertension | 1097 (52.2) | 2404 (54.6) | 0.068 |
| Missing | 23 | 74 |  |
| History of hyperlipidemia | 655 (32.2) | 1324 (30.8) | 0.294 |
| Missing | 91 | 183 |  |
| History of cancer | 232 (11.0) | 634 (14.2) | <0.001 |
| Missing | 10 | 18 |  |

IQR = interquartile range; TNM = tumor, lymph node, and metastasis staging system; NSAIDs = nonsteroidal anti-inflammatory drugs. ^1^ Sex-specific categories of abstainers, light, moderate, or heavy drinkers: for women, 0, >0–12, >12–25, or >25 g ethanol/day; for men, 0, >0–24, >24–50, or >50 g ethanol/day. ^1^Lifetime average metabolic equivalent of task hours/week.

**Table S3: Association between self-reported BMI at diagnosis or 5-14 years before diagnosis and mortality risk additionally adjusted for weight loss**

|  | **aHR (95% CI)^1^** | | |
| --- | --- | --- | --- |
|  | **Overall mortality** | **Cancer-related mortality** | **Non-CRC-related mortality** |
| BMI at diagnosis | | | |
| per 5 units | 0.93 (0.85, 1.00) | 0.92 (0.82, 1.03) | 0.95 (0.85, 1.07) |
| <20 | 1.35 (1.01, 1.79) | 1.11 (0.74, 1.67) | 1.55 (1.01, 2.36) |
| 20-25 | 1 | 1 | 1 |
| 25-30 | 0.94 (0.82, 1.07) | 0.96 (0.80, 1.17) | 0.90 (0.74, 1.11) |
| ≥30 | 0.88 (0.73, 1.05) | 0.85 (0.66, 1.11) | 0.96 (0.74, 1.24) |
| BMI 5-14 years before diagnosis | | | |
| per 5 units | 0.97 (0.90, 1.05) | 1.01 (0.90, 1.13) | 0.94 (0.84, 1.06) |
| <20 | 0.81 (0.50, 1.32) | 0.78 (0.42, 1.45) | 0.72 (0.31, 1.66) |
| 20-25 | 1 | 1 | 1 |
| 25-30 | 0.90 (0.78, 1.03) | 0.91 (0.75, 1.11) | 0.84 (0.68, 1.03) |
| ≥30 | 0.98 (0.82, 1.17) | 1.08 (0.85, 1.38) | 0.87 (0.67, 1.12) |

The multivariable Cox model was adjusted for age, sex, tumor stage, tumor location, treatment with chemotherapy or radiotherapy, alcohol consumption, physical activity, smoking status, regular statin use, nonsteroidal anti-inflammatory drug use, history of cardiovascular diseases, hyperlipidemia, hypertension, other cancers, and weight loss compared with weight 5-14 years before diagnosis and weight at diagnosis.

**Table S4: Associations between methylation-based BMI scores and CRC prognosis by BMI groups at diagnosis and by weight loss groups**

|  |  | **aHR (95%CI)^1^** | | |
| --- | --- | --- | --- | --- |
|  |  | **Overall mortality** | **CRC-related mortality** | **Non-CRC-related mortality** |
|  | **BMI at diagnosis groups** | | | |
| Mendelson et al. 2017 (mBMI-135) | <20 | 1.33 (0.89, 2.00) | 1.50 (0.82, 2.75) | 1.45 (0.72, 2.92) |
|  | 20-25 | 1.21 (1.08, 1.35) | 1.14 (0.98, 1.33) | 1.32 (1.11, 1.56) |
|  | 25-30 | 1.18 (1.07, 1.30) | 1.27 (1.10, 1.45) | 1.20 (1.04, 1.39) |
|  | ≥30 | 1.17 (1.01, 1.36) | 1.30 (1.04, 1.63) | 1.12 (0.90, 1.38) |
|  | *P_interaction_* | 0.09 | 0.062 | 0.315 |
| McCartney et al. 2018 (mBMI-1109) | <20 | 1.15 (0.81, 1.63) | 1.33 (0.76, 2.31) | 1.05 (0.61, 1.79) |
|  | 20-25 | 1.11 (0.99, 1.24) | 1.12 (0.96, 1.29) | 1.14 (0.97, 1.35) |
|  | 25-30 | 1.10 (1.00, 1.21) | 1.22 (1.06, 1.39) | 1.04 (0.91, 1.19) |
|  | ≥30 | 1.16 (0.99, 1.36) | 1.18 (0.93, 1.49) | 1.25 (1.00, 1.57) |
|  | *P_interaction_* | 0.097 | 0.093 | 0.414 |
| Hamilton et al. 2019 (mBMI-435) | <20 | 1.20 (0.83, 1.73) | 1.43 (0.83, 2.46) | 0.96 (0.55, 1.70) |
|  | 20-25 | 1.07 (0.96, 1.20) | 1.08 (0.93, 1.26) | 1.16 (0.99, 1.36) |
|  | 25-30 | 1.13 (1.03, 1.24) | 1.21 (1.06, 1.39) | 1.08 (0.94, 1.24) |
|  | ≥30 | 1.20 (1.03, 1.40) | 1.16 (0.92, 1.46) | 1.30 (1.04, 1.61) |
|  | *P_interaction_* | 0.058 | 0.038 | 0.293 |
| Do et al. 2022 (mBMI-397) | <20 | 1.43 (0.99, 2.05) | 1.29 (0.80, 2.08) | 1.93 (1.00, 3.72) |
|  | 20-25 | 1.21 (1.10, 1.34) | 1.24 (1.08, 1.42) | 1.22 (1.04, 1.43) |
|  | 25-30 | 1.21 (1.10, 1.33) | 1.32 (1.15, 1.51) | 1.11 (0.96, 1.28) |
|  | ≥30 | 1.13 (0.97, 1.32) | 1.16 (0.93, 1.45) | 1.15 (0.92, 1.43) |
|  | *P_interaction_* | 0.171 | 0.461 | 0.29 |
| Merzbacher et al. 2023 (mBMI-3506) | <20 | 0.98 (0.72, 1.34) | 0.84 (0.54, 1.28) | 1.33 (0.77, 2.29) |
|  | 20-25 | 0.99 (0.89, 1.11) | 0.94 (0.81, 1.10) | 1.13 (0.96, 1.33) |
|  | 25-30 | 1.06 (0.96, 1.17) | 1.20 (1.04, 1.39) | 0.99 (0.87, 1.12) |
|  | ≥30 | 1.10 (0.93, 1.29) | 1.19 (0.92, 1.54) | 1.03 (0.82, 1.29) |
|  | *P_interaction_* | 0.315 | 0.059 | 0.3 |
|  | **Weight loss groups** |  |  |  |
| Mendelson et al. 2017 (mBMI-135) | No weight loss | 1.14 (1.00, 1.29) | 1.32 (1.16, 1.51) | 1.19 (1.09, 1.30) |
|  | Loss 2-5 kg | 1.51 (1.16, 1.96) | 1.06 (0.82, 1.37) | 1.27 (1.07, 1.51) |
|  | Loss > 5kg | 1.16 (1.01, 1.33) | 1.07 (0.91, 1.26) | 1.13 (1.02, 1.26) |
|  | *P_interaction_* | 0.323 | 0.143 | 0.037 |
| McCartney et al. 2018 (mBMI-1109) | No weight loss | 1.12 (0.99, 1.27) | 1.15 (1.01, 1.32) | 1.11 (1.01, 1.21) |
|  | Loss 2-5 kg | 1.26 (0.97, 1.63) | 1.24 (0.97, 1.59) | 1.16 (0.97, 1.38) |
|  | Loss > 5kg | 1.08 (0.94, 1.25) | 0.96 (0.81, 1.14) | 1.04 (0.94, 1.16) |
|  | *P_interaction_* | 0.576 | 0.553 | 0.193 |
| Hamilton et al. 2019 (mBMI-435) | No weight loss | 1.14 (1.00, 1.29) | 1.24 (1.08, 1.41) | 1.14 (1.04, 1.24) |
|  | Loss 2-5 kg | 1.26 (0.98, 1.61) | 1.21 (0.96, 1.53) | 1.18 (1.00, 1.39) |
|  | Loss > 5kg | 1.05 (0.91, 1.23) | 0.92 (0.78, 1.08) | 1.01 (0.90, 1.12) |
|  | *P_interaction_* | 0.095 | 0.361 | 0.015 |
| Do et al. 2022 (mBMI-397) | No weight loss | 1.22 (1.08, 1.38) | 1.22 (1.07, 1.40) | 1.19 (1.08, 1.30) |
|  | Loss 2-5 kg | 1.28 (1.02, 1.60) | 1.04 (0.81, 1.33) | 1.15 (0.98, 1.36) |
|  | Loss > 5kg | 1.21 (1.05, 1.39) | 1.00 (0.85, 1.18) | 1.15 (1.04, 1.28) |
|  | *P_interaction_* | 0.629 | 0.614 | 0.108 |
| Merzbacher et al. 2023 (mBMI-3506) | No weight loss | 1.09 (0.95, 1.25) | 1.12 (0.98, 1.28) | 1.08 (0.98, 1.18) |
|  | Loss 2-5 kg | 1.09 (0.86, 1.39) | 1.10 (0.87, 1.41) | 1.07 (0.91, 1.26) |
|  | Loss > 5kg | 1.01 (0.87, 1.18) | 0.92 (0.78, 1.08) | 0.97 (0.87, 1.08) |
|  | *P_interaction_* | 0.459 | 0.751 | 0.133 |

BMI = body mass index; mBMI = methylation-based BMI, the number after mBMI stands for the number of CpGs included in the score; aHR = adjusted hazards ratio; CI = confidence interval.

^1^The HR represent the hazard ratio per 5-unit increase in BMI, and per 1 standard deviation increase for the five methylation-based BMI scores. The multivariable Cox model was adjusted for age, sex, tumor stage, tumor location, treatment with chemotherapy or radiotherapy, alcohol consumption, physical activity, smoking status, regular statin use, nonsteroidal anti-inflammatory drug use, history of cardiovascular diseases, hyperlipidemia, hypertension, and other cancer

**Table S5: Subgroup analyses for the associations between self-reported BMI and blood methylation-based BMI scores and CRC prognosis**

|  |  | **aHR (95%CI)^1^** | | |
| --- | --- | --- | --- | --- |
|  | **Subgroup** | **Overall mortality** | **CRC-related mortality** | **Non-CRC-related mortality** |
|  | **Age** | | | |
| BMI at diagnosis | <70 yr | 0.89 (0.79, 1.00) | 0.86 (0.74, 1.00) | 0.93 (0.77, 1.14) |
|  | ≥70yr | 0.91 (0.82, 1.01) | 0.97 (0.83, 1.14) | 0.88 (0.76, 1.01) |
|  | *P_interaction_* | 0.682 | 0.829 | 0.328 |
| Mendelson et al. 2017 (mBMI-135) | <70 yr | 1.21 (1.09, 1.33) | 1.23 (1.09, 1.39) | 1.15 (0.97, 1.36) |
|  | ≥70yr | 1.18 (1.09, 1.29) | 1.15 (1.02, 1.30) | 1.20 (1.07, 1.34) |
|  | *P_interaction_* | 0.453 | 0.254 | 0.9 |
| McCartney et al. 2018 (mBMI-1109) | <70 yr | 1.05 (0.95, 1.16) | 1.10 (0.97, 1.24) | 0.96 (0.81, 1.13) |
|  | ≥70yr | 1.16 (1.07, 1.26) | 1.17 (1.04, 1.33) | 1.13 (1.01, 1.27) |
|  | *P_interaction_* | 0.442 | 0.86 | 0.219 |
| Hamilton et al. 2019 (mBMI-435) | <70 yr | 1.09 (0.99, 1.21) | 1.10 (0.97, 1.25) | 1.06 (0.90, 1.25) |
|  | ≥70yr | 1.12 (1.03, 1.22) | 1.15 (1.01, 1.30) | 1.11 (1.00, 1.24) |
|  | *P_interaction_* | 0.856 | 0.919 | 0.885 |
| Do et al. 2022  (mBMI-397) | <70 yr | 1.11 (1.01, 1.23) | 1.19 (1.05, 1.35) | 0.98 (0.83, 1.16) |
|  | ≥70yr | 1.23 (1.14, 1.33) | 1.24 (1.10, 1.40) | 1.17 (1.05, 1.30) |
|  | *P_interaction_* | 0.539 | 0.92 | 0.136 |
| Merzbacher et al. 2023 (mBMI-3506) | <70 yr | 1.03 (0.94, 1.14) | 1.07 (0.94, 1.21) | 0.95 (0.81, 1.13) |
|  | ≥70yr | 1.04 (0.95, 1.13) | 1.01 (0.88, 1.17) | 1.04 (0.93, 1.16) |
|  | *P_interaction_* | 0.543 | 0.343 | 0.74 |
|  | **Sex** |  |  |  |
| BMI at diagnosis | Female | 0.84 (0.75, 0.94) | 0.88 (0.76, 1.03) | 0.80 (0.67, 0.96) |
|  | Male | 0.93 (0.84, 1.03) | 0.91 (0.78, 1.06) | 0.95 (0.82, 1.10) |
|  | *P_interaction_* | 0.317 | 0.872 | 0.274 |
| Mendelson et al. 2017 (mBMI-135) | Female | 1.17 (1.05, 1.31) | 1.24 (1.07, 1.43) | 1.12 (0.95, 1.33) |
|  | Male | 1.19 (1.10, 1.29) | 1.18 (1.06, 1.32) | 1.21 (1.08, 1.35) |
|  | *P_interaction_* | 0.94 | 0.362 | 0.599 |
| McCartney et al. 2018 (mBMI-1109) | Female | 1.04 (0.94, 1.16) | 1.13 (0.99, 1.30) | 0.96 (0.82, 1.12) |
|  | Male | 1.12 (1.03, 1.21) | 1.14 (1.02, 1.28) | 1.12 (0.99, 1.26) |
|  | *P_interaction_* | 0.65 | 0.63 | 0.268 |
| Hamilton et al. 2019 (mBMI-435) | Female | 1.03 (0.93, 1.14) | 1.09 (0.95, 1.26) | 0.99 (0.85, 1.15) |
|  | Male | 1.13 (1.04, 1.22) | 1.14 (1.02, 1.28) | 1.16 (1.03, 1.30) |
|  | *P_interaction_* | 0.47 | 0.908 | 0.172 |
| Do et al. 2022  (mBMI-397) | Female | 1.06 (0.95, 1.18) | 1.11 (0.97, 1.28) | 1.00 (0.85, 1.17) |
|  | Male | 1.24 (1.15, 1.34) | 1.30 (1.17, 1.45) | 1.15 (1.02, 1.29) |
|  | *P_interaction_* | 0.286 | 0.487 | 0.461 |
| Merzbacher et al. 2023 (mBMI-3506) | Female | 0.96 (0.86, 1.07) | 1.00 (0.86, 1.16) | 0.94 (0.80, 1.10) |
|  | Male | 1.05 (0.97, 1.14) | 1.07 (0.95, 1.20) | 1.03 (0.92, 1.16) |
|  | *P_interaction_* | 0.502 | 0.968 | 0.55 |
|  | **TNM stage** | | | |
| BMI at diagnosis | I/II | 0.78 (0.62, 0.99) | 0.95 (0.83, 1.09) | 0.90 (0.80, 1.01) |
|  | III/IV | 0.95 (0.85, 1.08) | 0.82 (0.67, 1.00) | 0.92 (0.83, 1.01) |
|  | *P_interaction_* | 0.772 | 0.141 | 0.428 |
| Mendelson et al. 2017 (mBMI-135) | I/II | 1.12 (0.93, 1.36) | 1.24 (1.11, 1.39) | 1.20 (1.09, 1.32) |
|  | III/IV | 1.19 (1.08, 1.31) | 1.13 (0.96, 1.32) | 1.16 (1.07, 1.26) |
|  | *P_interaction_* | 0.185 | 0.80 | 0.241 |
| McCartney et al. 2018 (mBMI-1109) | I/II | 1.02 (0.85, 1.23) | 1.10 (0.98, 1.23) | 1.07 (0.97, 1.18) |
|  | III/IV | 1.16 (1.05, 1.28) | 1.04 (0.88, 1.22) | 1.11 (1.02, 1.21) |
|  | *P_interaction_* | 0.453 | 0.202 | 0.743 |
| Hamilton et al. 2019 (mBMI-435) | I/II | 1.07 (0.89, 1.29) | 1.15 (1.03, 1.28) | 1.12 (1.02, 1.23) |
|  | III/IV | 1.14 (1.03, 1.26) | 0.97 (0.83, 1.14) | 1.08 (0.99, 1.18) |
|  | *P_interaction_* | 0.617 | 0.404 | 0.191 |
| Do et al. 2022  (mBMI-397) | I/II | 1.02 (0.85, 1.22) | 1.14 (1.02, 1.27) | 1.11 (1.01, 1.22) |
|  | III/IV | 1.28 (1.16, 1.41) | 1.05 (0.89, 1.23) | 1.21 (1.11, 1.31) |
|  | *P_interaction_* | 0.096 | 0.053 | 0.536 |
| Merzbacher et al. 2023 (mBMI-3506) | I/II | 0.86 (0.72, 1.03) | 1.04 (0.93, 1.16) | 0.99 (0.90, 1.09) |
|  | III/IV | 1.11 (1.00, 1.23) | 0.96 (0.82, 1.14) | 1.06 (0.97, 1.16) |
|  | *P_interaction_* | 0.034 | 0.007 | 0.955 |
|  | **Metastasis** | | | |
| BMI at diagnosis | No | 0.90 (0.83, 0.98) | 0.88 (0.77, 1.01) | 0.93 (0.83, 1.04) |
|  | Yes | 0.93 (0.78, 1.10) | 0.99 (0.83, 1.18) | 0.22 (0.07, 0.67) |
|  | *P_interaction_* | 0.706 | 0.472 | 0.008 |
| Mendelson et al. 2017 (mBMI-135) | No | 1.17 (1.09, 1.26) | 1.19 (1.06, 1.33) | 1.18 (1.08, 1.30) |
|  | Yes | 1.23 (1.08, 1.41) | 1.22 (1.06, 1.41) | 1.30 (0.65, 2.57) |
|  | *P_interaction_* | 0.619 | 0.904 | 0.572 |
| McCartney et al. 2018 (mBMI-1109) | No | 1.09 (1.01, 1.17) | 1.11 (0.99, 1.24) | 1.08 (0.99, 1.19) |
|  | Yes | 1.21 (1.05, 1.39) | 1.22 (1.05, 1.41) | 1.01 (0.52, 1.93) |
|  | *P_interaction_* | 0.36 | 0.482 | 0.866 |
| Hamilton et al. 2019 (mBMI-435) | No | 1.11 (1.04, 1.19) | 1.14 (1.02, 1.27) | 1.11 (1.01, 1.21) |
|  | Yes | 1.15 (1.00, 1.32) | 1.14 (0.99, 1.32) | 1.11 (0.56, 2.21) |
|  | *P_interaction_* | 0.682 | 0.904 | 0.773 |
| Do et al. 2022  (mBMI-397) | No | 1.12 (1.04, 1.20) | 1.14 (1.03, 1.28) | 1.11 (1.01, 1.21) |
|  | Yes | 1.33 (1.16, 1.54) | 1.34 (1.16, 1.56) | 1.16 (0.61, 2.21) |
|  | *P_interaction_* | 0.006 | 0.091 | 0.496 |
| Merzbacher et al. 2023 (mBMI-3506) | No | 1.00 (0.93, 1.08) | 0.99 (0.88, 1.11) | 1.02 (0.93, 1.12) |
|  | Yes | 1.18 (1.02, 1.35) | 1.17 (1.01, 1.35) | 1.16 (0.59, 2.27) |
|  | *P_interaction_* | 0.007 | 0.041 | 0.5 |
|  | **CRC location** | | | |
| BMI at diagnosis | Distal colon | 0.85 (0.72, 0.99) | 0.85 (0.68, 1.06) | 0.85 (0.68, 1.08) |
|  | Proximal colon | 0.85 (0.75, 0.96) | 0.92 (0.77, 1.10) | 0.86 (0.73, 1.03) |
|  | Rectum | 0.94 (0.82, 1.07) | 0.96 (0.81, 1.14) | 0.90 (0.74, 1.11) |
|  | *P_interaction_* | 0.292 | 0.307 | 0.637 |
| Mendelson et al. 2017 (mBMI-135) | Distal colon | 1.18 (1.04, 1.34) | 1.29 (1.08, 1.55) | 1.12 (0.93, 1.34) |
|  | Proximal colon | 1.22 (1.10, 1.35) | 1.20 (1.03, 1.40) | 1.26 (1.10, 1.45) |
|  | Rectum | 1.16 (1.05, 1.29) | 1.18 (1.03, 1.35) | 1.14 (0.97, 1.35) |
|  | *P_interaction_* | 0.49 | 0.745 | 0.398 |
| McCartney et al. 2018 (mBMI-1109) | Distal colon | 1.10 (0.97, 1.25) | 1.12 (0.94, 1.35) | 1.13 (0.94, 1.35) |
|  | Proximal colon | 1.14 (1.03, 1.27) | 1.16 (0.99, 1.35) | 1.18 (1.02, 1.36) |
|  | Rectum | 1.04 (0.94, 1.16) | 1.14 (0.99, 1.30) | 0.90 (0.76, 1.07) |
|  | *P_interaction_* | 0.448 | 0.472 | 0.12 |
| Hamilton et al. 2019 (mBMI-435) | Distal colon | 1.14 (1.01, 1.29) | 1.13 (0.94, 1.35) | 1.18 (0.98, 1.42) |
|  | Proximal colon | 1.13 (1.02, 1.25) | 1.12 (0.96, 1.31) | 1.18 (1.03, 1.36) |
|  | Rectum | 1.04 (0.94, 1.16) | 1.14 (0.99, 1.31) | 0.92 (0.78, 1.08) |
|  | *P_interaction_* | 0.521 | 0.501 | 0.063 |
| Do et al. 2022  (mBMI-397) | Distal colon | 1.24 (1.10, 1.40) | 1.34 (1.13, 1.58) | 1.10 (0.92, 1.32) |
|  | Proximal colon | 1.19 (1.07, 1.32) | 1.14 (0.98, 1.34) | 1.23 (1.06, 1.41) |
|  | Rectum | 1.13 (1.03, 1.25) | 1.24 (1.09, 1.41) | 0.96 (0.81, 1.13) |
|  | *P_interaction_* | 0.537 | 0.379 | 0.246 |
| Merzbacher et al. 2023 (mBMI-3506) | Distal colon | 1.05 (0.92, 1.19) | 1.09 (0.90, 1.31) | 1.04 (0.88, 1.24) |
|  | Proximal colon | 1.03 (0.92, 1.14) | 1.05 (0.89, 1.23) | 1.05 (0.91, 1.22) |
|  | Rectum | 0.99 (0.88, 1.10) | 1.03 (0.89, 1.20) | 0.93 (0.78, 1.11) |
|  | *P_interaction_* | 0.818 | 0.715 | 0.462 |
|  | **Blood collection time** | | | |
| BMI at diagnosis | <3 months | 0.90 (0.82, 0.99) | 0.87 (0.76, 0.99) | 0.98 (0.84, 1.13) |
|  | ≥3 months | 0.87 (0.76, 0.99) | 0.98 (0.82, 1.17) | 0.79 (0.66, 0.96) |
|  | *P_interaction_* | 0.906 | 0.263 | 0.148 |
| Mendelson et al. 2017 (mBMI-135) | <3 months | 1.16 (1.08, 1.26) | 1.17 (1.05, 1.31) | 1.16 (1.03, 1.31) |
|  | ≥3 months | 1.16 (1.05, 1.28) | 1.16 (1.00, 1.34) | 1.18 (1.02, 1.35) |
|  | *P_interaction_* | 0.675 | 0.946 | 0.552 |
| McCartney et al. 2018 (mBMI-1109) | <3 months | 1.07 (0.99, 1.16) | 1.09 (0.98, 1.21) | 1.06 (0.94, 1.20) |
|  | ≥3 months | 1.09 (0.99, 1.21) | 1.16 (1.00, 1.35) | 1.04 (0.90, 1.21) |
|  | *P_interaction_* | 0.726 | 0.456 | 0.91 |
| Hamilton et al. 2019 (mBMI-435) | <3 months | 1.06 (0.98, 1.14) | 1.10 (0.98, 1.22) | 1.04 (0.93, 1.18) |
|  | ≥3 months | 1.11 (1.00, 1.23) | 1.11 (0.95, 1.29) | 1.14 (0.99, 1.32) |
|  | *P_interaction_* | 0.423 | 0.833 | 0.33 |
| Do et al. 2022  (mBMI-397) | <3 months | 1.14 (1.05, 1.23) | 1.15 (1.03, 1.28) | 1.08 (0.96, 1.22) |
|  | ≥3 months | 1.18 (1.07, 1.31) | 1.30 (1.13, 1.50) | 1.06 (0.91, 1.24) |
|  | *P_interaction_* | 0.284 | 0.05 | 0.926 |
| Merzbacher et al. 2023 (mBMI-3506) | <3 months | 0.99 (0.91, 1.07) | 1.00 (0.89, 1.13) | 0.99 (0.88, 1.11) |
|  | ≥3 months | 1.04 (0.94, 1.16) | 1.10 (0.94, 1.28) | 1.01 (0.88, 1.17) |
|  | *P_interaction_* | 0.349 | 0.266 | 0.844 |
|  | **Timing of blood collection relative to neo-/adjuvant chemotherapy** | | | |
| BMI at diagnosis | Before/No | 0.91 (0.83, 1.00) | 0.90 (0.78, 1.03) | 0.96 (0.84, 1.08) |
|  | After | 0.82 (0.71, 0.95) | 0.92 (0.77, 1.09) | 0.68 (0.52, 0.91) |
|  | *P_interaction_* | 0.432 | 0.918 | 0.194 |
| Mendelson et al. 2017 (mBMI-135) | Before/No | 1.25 (1.16, 1.35) | 1.24 (1.11, 1.38) | 1.27 (1.14, 1.41) |
|  | After | 1.02 (0.90, 1.15) | 1.11 (0.96, 1.29) | 0.91 (0.73, 1.13) |
|  | *P_interaction_* | 0.001 | 0.095 | 0.007 |
| McCartney et al. 2018 (mBMI-1109) | Before/No | 1.14 (1.06, 1.23) | 1.19 (1.06, 1.34) | 1.14 (1.02, 1.26) |
|  | After | 0.99 (0.88, 1.11) | 1.06 (0.92, 1.22) | 0.88 (0.71, 1.10) |
|  | *P_interaction_* | 0.061 | 0.172 | 0.136 |
| Hamilton et al. 2019 (mBMI-435) | Before/No | 1.12 (1.04, 1.21) | 1.14 (1.02, 1.28) | 1.17 (1.06, 1.29) |
|  | After | 1.01 (0.90, 1.13) | 1.10 (0.95, 1.26) | 0.87 (0.71, 1.08) |
|  | *P_interaction_* | 0.179 | 0.514 | 0.076 |
| Do et al. 2022  (mBMI-397) | Before/No | 1.23 (1.14, 1.32) | 1.23 (1.11, 1.38) | 1.20 (1.08, 1.33) |
|  | After | 1.06 (0.95, 1.19) | 1.25 (1.09, 1.43) | 0.77 (0.62, 0.96) |
|  | *P_interaction_* | 0.177 | 0.825 | 0.004 |
| Merzbacher et al. 2023 (mBMI-3506) | Before/No | 1.03 (0.95, 1.11) | 1.01 (0.89, 1.14) | 1.07 (0.97, 1.18) |
|  | After | 0.98 (0.87, 1.11) | 1.08 (0.93, 1.25) | 0.80 (0.64, 0.99) |
|  | *P_interaction_* | 0.817 | 0.429 | 0.179 |
|  | **Timing of blood collection relative to neo-/adjuvant chemoradiotherapy** | | | |
| BMI at diagnosis | Before/No | 0.90 (0.82, 0.99) | 0.89 (0.77, 1.03) | 0.96 (0.84, 1.08) |
|  | After | 0.85 (0.74, 0.97) | 0.92 (0.78, 1.09) | 0.75 (0.58, 0.97) |
|  | *P_interaction_* | 0.738 | 0.728 | 0.302 |
| Mendelson et al. 2017 (mBMI-135) | Before/No | 1.25 (1.16, 1.35) | 1.25 (1.12, 1.40) | 1.25 (1.13, 1.39) |
|  | After | 1.03 (0.92, 1.16) | 1.11 (0.96, 1.28) | 0.97 (0.79, 1.19) |
|  | *P_interaction_* | 0.001 | 0.112 | 0.02 |
| McCartney et al. 2018 (mBMI-1109) | Before/No | 1.14 (1.05, 1.23) | 1.21 (1.07, 1.36) | 1.11 (1.00, 1.23) |
|  | After | 1.01 (0.90, 1.13) | 1.05 (0.92, 1.21) | 0.99 (0.81, 1.21) |
|  | *P_interaction_* | 0.125 | 0.165 | 0.446 |
| Hamilton et al. 2019 (mBMI-435) | Before/No | 1.12 (1.04, 1.21) | 1.15 (1.03, 1.30) | 1.16 (1.04, 1.28) |
|  | After | 1.03 (0.92, 1.15) | 1.10 (0.95, 1.26) | 0.94 (0.78, 1.15) |
|  | *P_interaction_* | 0.235 | 0.502 | 0.172 |
| Do et al. 2022  (mBMI-397) | Before/No | 1.24 (1.15, 1.33) | 1.25 (1.12, 1.40) | 1.20 (1.08, 1.33) |
|  | After | 1.06 (0.95, 1.19) | 1.24 (1.08, 1.42) | 0.81 (0.66, 1.00) |
|  | *P_interaction_* | 0.125 | 0.932 | 0.004 |
| Merzbacher et al. 2023 (mBMI-3506) | Before/No | 1.03 (0.95, 1.11) | 1.01 (0.89, 1.15) | 1.07 (0.96, 1.18) |
|  | After | 0.99 (0.88, 1.12) | 1.08 (0.94, 1.25) | 0.85 (0.69, 1.05) |
|  | *P_interaction_* | 0.759 | 0.371 | 0.214 |

BMI = body mass index; mBMI = methylation-based BMI, the number after mBMI stands for the number of CpGs included in the score; aHR = adjusted hazards ratio; CI = confidence interval.

^1^The HR represent the hazard ratio per 5-unit increase in BMI, and per 1 standard deviation increase for the five methylation-based BMI scores. The multivariable Cox model was adjusted for age, sex, tumor stage, tumor location, treatment with chemotherapy or radiotherapy, alcohol consumption, physical activity, smoking status, regular statin use, nonsteroidal anti-inflammatory drug use, history of cardiovascular diseases, hyperlipidemia, hypertension, and other cancer

**Fig. S2: Spearman correlation coefficients between blood DNA methylation-based BMI scores and historical self-reported BMI among patients whose weight change from 5-14 years ago to diagnosis >5/kg (*N* = 935)**

BMI = body mass index; mBMI = methylation-based BMI. The number after mBMI stands for the number of CpGs in the score, and the superscript represents the reference of the paper that developed this score. For the Spearman correlation coefficients, all *p* values were <0.0001.

**Fig. S3: Gene Ontology enrichment analysis for Mendelson et al. 2017 DNA methylation score (mBMI-135)**

**Fig. S4: Gene Ontology enrichment analysis for Do et al. 2022 DNA methylation score (mBMI-397)**

**Fig. S5: Gene Ontology enrichment analysis for Hamilton et al. 2019 DNA methylation score (mBMI-435)**

**Fig. S6: Gene Ontology enrichment analysis for McCartney et al. 2018 DNA methylation score (mBMI-1109)**

**Fig. S7: Gene Ontology enrichment analysis for Merzbacher et al. 2023 DNA methylation score (mBMI-3506).**
